# Changes in risk factors for young male suicide in Newcastle upon Tyne, 1961–2009

**DOI:** 10.1192/pb.bp.114.048884

**Published:** 2016-06

**Authors:** Keith R. Linsley, Martin A. Schapira, Kurt Schapira, Clare Lister

**Affiliations:** 1 Tees, Esk and Wear Valleys NHS Foundation Trust; 2 Newcastle University

## Abstract

**Aims and method**

To ascertain differences in patterns of suicide in young men over three decades (1960s, 1990s and 2000s) and discuss implications for suicide prevention. Data on suicides and open verdicts in men aged 15–34 were obtained from coroner's records in Newcastle upon Tyne and analysed using SPSS software.

**Results**

An increase in suicide rates from the first to the second decade was followed by a fall in the third decade. This was associated with an increasing proportion of single men, those living alone, unemployment, consumption of alcohol, use of hanging, previous suicide attempt and history of treatment for mental illness.

**Clinical implications**

This study highlights the need for more interventions and focus to be given to young males in the suicide prevention area and is of high importance in the field of public health. Areas that could be tackled include reducing access to means of suicide, reducing alcohol use, support for relationship difficulties, engagement with mental health services and management of chronic illness.

In a previous study in Newcastle upon Tyne, England,^1^ we reported on the suicide profile of the population over two periods, 1961–65 and 1985–94, and showed a dramatic fall of suicide in women and a modest decline in men. The only group who failed to share this trend were young men (aged 15–34), in whom the suicide rate increased, a trend also reported in other studies.^2,3^ We found that relative risk of suicide (based on population data) decreased between the two periods studied for divorced men of all ages (8.36 to 2.86), unemployed men of all ages (9.57 to 2.34) and both men and women living alone (all ages; 11.91 to 4.34), whereas the relative risk for being single increased for men and women of all ages (0.69 to 1.46).^1^

The aim of the present investigation was to examine this group of young males in greater detail and over an extended time period. In addition, suicide prevention strategies have become a focus of government intervention from 1999,^4^ and we wished to find out whether anything new was emerging in a new century and identify any changes in characteristics associated with suicide.

Examination of coroner's records permits a more detailed scrutiny of factors associated with a person's death and can yield information not available from studies based on data supplied to the Office for National Statistics, including analysis of which open verdicts should (or should not) be included. Local studies, such as we describe, may become increasingly important as coroners can now provide narrative verdicts, but such verdicts may prove difficult for national data collection centres to interpret in the context of deciding whether they will be included in future suicide statistics.

## Method

The study focused on men aged 15–34 for whom a verdict of suicide was recorded by Newcastle coroners during three 10-year periods: 1961–1970 (period A), 1990–1999 (period B) and 2000–2009 (period C). Coroners only record a verdict of suicide when this is beyond reasonable doubt and otherwise can record an open or undetermined verdict. We established from previous suicide research that it is important to examine cases which received such open or undetermined verdicts^5^ to assess which of these, on the balance of probabilities, could or should be included as suicides for the purpose of research. Thus, two authors established deaths as probable suicides based on information within the coroner's record. If they remained uncertain then a consensus was reached between them and a third author based on a balance of probabilities. Factors taken into account included third-party descriptions of: mental state near to the person's time of death, their past mental illness, nature and degree of planning of a method of death, nature of any precipitating factor to death, and autopsy findings. As two authors examined records for all time periods an equal standard was applied across all decades. Narrative verdicts, which do not specify suicide or open verdict, were not found in any of the records during this study.

We obtained age-specific male population statistics for Newcastle for the three decades to calculate rates of suicide. We hoped population statistics would be available that broke this information down into categories such as unemployment, marital status and living status, but neither the Office for National Statistics, nor Newcastle County Council or other sources had such information.

Only cases where the deceased person was officially resident within Newcastle upon Tyne city boundaries at the time of death were included. This was to allow comparison of the data with appropriate city demographic data and to exclude records of non-residents who were transferred to a regional medical centre within the city for treatment and later died. Residents of Newcastle who died by suicide outside the area of the city (and whose inquests were therefore held elsewhere) were not included. In the previous study^1^ we calculated that there were on average 1.5 men and 0.9 women who died by suicide outside the area per year.

We used the term ‘precipitating factor’ to denote the main apparent reason for suicide as it emerged in the context of the history recorded from statements by relatives and close friends, often substantiated by a suicide note. Data were recorded on semi-structured forms and transferred to an SPSS database for analysis. The procedure of extracting data from the coroner's records was the same for all three periods. Data for each factor were analysed using χ^2^-tests, with a significance level of 0.05. A logistical regression analysis (multinominal analysis) was then completed for all factors found to be statistically significant.

## Results

There were 49 cases of suicide of young men during period A (population of males aged 15–34 was 35 390)^6^ and 92 in period B (population of males aged 15–34 was 38 314),^7^ giving an increase in the suicide rate from to 13.85 to 24.01 per 100 000 per year of an age-adjusted population. This was followed by a fall to 60 cases in period C (population of males aged 15–34, 40 914),^8^ equating to a rate of 14.66 per 100 000 per year.

As can be seen in Table 1, there has been a significant increase in the proportion of single men in each group, from 63% in period A to 66% in period B and 87% in period C.

**Table 1:** Suicide findings within Newcastle upon Tyne over three decades

Factor	Period A (1961–1970)	Period B (1990–1999)	Period C (2000–2009)			
				
		*n* (%)		χ^2^	*P*	d.f.
Single	31 (63.3)	61 (66.6)	52 (86.7)	18.4	<0.01	2

Living alone	13 (26.5)	35 (38)	29 (48.3)	5.43	0.066	2

Alcohol at autopsy	10 (20.4)	38 (41.3)	32 (53.3)	12.36	0.002	2

Unemployed	14 (28.6)	38 (41.3)	40 (66.7)	16.74	<0.001	2

Evidence of precipitating factor	30 (61.2)	65 (70.7)	35 (58.3)	2.75	0.09	2

Never received psychiatric treatment	26 (53.1)	43 (46.7)	14 (23.3)	13	<0.05	2

Previous suicide attempt						
0	32 (65.3)	58 (63.0)	20 (33.3)	22.1	<0.01	4
>1	3 (6.1)	20 (21.7)	17 (28.8)			

Moderate/severe physical health problem	8 (16.3)	12 (13.3)	1 (1.7)	7.27	0.026	2

### Living arrangements

Only 27% of the men in the 1960s were living alone, rising to 38% in the 1990s and 48% in the 2000s (Table 1). Only one person was homeless across the first two periods and there were none in period C. None of the men were in shared living or sheltered accommodation in period A but there were 8 (9%) in period B and 4 (7%) in period C.

### The role of alcohol

There was a rise in alcohol detected at autopsy over the three periods, from 20% to 41% to 53% (Table 1). The number of those classified as intoxicated also rose from 5 (10%) in period A, to 16 (17%) in period B and 15 (25%) in period C.

### Employment

Rates of unemployment in the young men rose from 29% in the 1960s to 41% in the 1990s to 67% in the 2000s (Table 1).

### Previous treatment of mental illness

We noticed a fall in the numbers of young men who had never received psychiatric treatment from either their general practitioner (GP) or mental health services: from 53% in the 1960s, to 47% in the 1990s and finally, to 23% in the 2000s (Table 1). As regards contact with psychiatric services, only 3 men in period A (6%) had received treatment on an out-patient basis, compared with 14 in period B (16%) and 13 in period C (22%). In period A, 15 young men (31%) had at some time received in-patient treatment; this was 19 (22%) in period B and 16 (26%) in period C. There were no cases awaiting admission at the time of the suicide.

### Previous suicide attempts

There was no history of a suicide attempt in 65% of cases in period A, 63% in period B and 33% in period C. Those with more than one attempt rose from 6% to 22% and 29% in the three periods (Table 1).

### Physical health

If the coroner's records identified a pre-existing medical condition, a subjective judgement was made as to whether the effect on the individual was mild, moderate or severe based on witness statements and medical reports (Table 1). This seemed to play little role in the deaths we reviewed.

### Mode of suicide

As seen in Table 2, the majority of people used overdose of solids or gases as a method of suicide in period A. In period B there had been an increase in the use of violent methods and a change in the substances taken in overdose. There was an increase in the role of antidepressants used as a suicide method and this continued into period C, where the antidepressants were all selective serotonin reuptake inhibitors (SSRI) except in one person who took a tricyclic antidepressant. Only in three cases could death be solely attributed to the SSRI since other drugs were taken at the same time, a feature reflected by an increase in ‘drug combinations’ in this period.

**Table 2:** Methods of suicide within Newcastle upon Tyne over three decades^a^

Method	Period A (*n*=49) (1961–1970)	Period B (*n*=92) (1990–1999)	Period C (*n*=60) (2000–2009)
	
		*n* (%)	
Carbon monoxide	13 (26)	11 (12)	1 (2)

Barbiturates	13 (26)	0	0

Antidepressants	1 (2)	10 (11)	9 (15)

Anxiolytics	0	7 (8)	5 (8)

Salicylates	3 (6)	0	0

Paracetamol	1 (2)	8 (9)	6 (10)

Other substances	3 (6)	13 (14)	22 (37)

Drug combination	3 (6)	11 (12)	16 (27)

Hanging	2 (4)	34 (37)	29 (48)

Jumping	8 (16)	12 (13)	9 (15)

Drowning	6 (12)	5 (5)	1 (2)

Other	5 (10)	6 (7)	1 (2)

a. People can be counted in more than one category if a combination of methods were used.

There has also been a notable rise in ‘other substances’ which include prescribed medications, over-the-counter drugs and illicit drugs. As the numbers were significantly greater in period C than in two earlier periods, we subsequently examined precisely which drugs were used. These included heroin (4 cases), cocaine (5 cases), methadone (2 cases), cannabis (2 cases), ecstasy (1 case), benzodiazepines (3 cases), codeine (2 cases) and co-proxamol (3 cases).

### Precipitating factors

As seen in Table 1, an evident precipitating factor to suicide was present in 61% of deaths in period A, 71% in period B and 58% in period C. These occurred with similar frequency in the three cohorts with the exception of ‘failure of a long-term relationship’, which in period A accounted for only 4 cases (8%), rising to 20 (22%) and 13 cases (22%) in period B and C (Table 3). There was a drop in physical illness, court appearance and housing issues (‘domiciliary anxiety’) being identified as precipitating factors across the three periods (Table 3).

**Table 3:** Principal precipitating factors within Newcastle upon Tyne over three decades

	Period A (1961–1970)	Period B (1990–1999)	Period C (2000–2009)		
			
Precipitating factor		*n* (%)		χ^2^	*P*
Physical illness	5 (10.2)	7 (7.6)	0 (0.0)	5.81	0.06

Failure of long-term relationship	4 (8.2)	20 (21.7)	13 (21.7)	4.60	0.10

Bereavement	2 (4.1)	6 (6.5)	1 (1.7)	2.02	0.36

Court appearance	6 (12.2)	8 (8.7)	4 (6.7)	1.04	0.59

Cohabital strife	10 (20.4)	8 (8.7)	2 (3.3)	10.59	0.03

Awaiting hospital	1 (2.0)	0 (0.0)	2 (3.3)	2.88	0.24

Domicillary anxiety	5 (10.2)	7 (7.6)	1 (1.7)	3.13	0.21

Other	8 (16.3)	20 (21.7)	14 (23.3)	0.87	0.65

Combination	5 (10.2)	11 (12.0)	4 (6.7)	1.14	0.51

### Logistical regression analysis

Multinominal logistical regression analysis was performed to establish any adjusted association between multiple risk factors and the likelihood of the suicide event occurring in one of the time periods. We included the six factors found to be significantly different (*P*<0.05) between the three periods (being single, alcohol present at autopsy, unemployment, having never received psychiatric treatment, previous suicide attempt, and moderate or severe physical health problem).

Statistically significant relationships in this model to predict suicide were being unemployed (period A to period C odds ratio (OR)=0.098, *P*<0.001; period B to period C OR=0.36, *P*=0.039), being single (period A to period C OR=0.079, *P*=0.005; period B to period C OR=0.123, *P*=0.011) and having a moderate or severe physical illness (period A to period C OR=54.66, *P*=0.001; period B to period C=11.62, *P*=0.031). Previous suicide attempt was only significant in the final model for period A compared with period C and not for period B compared with period C (OR=7.41, *P*=0.049).

## Discussion

A striking rise and subsequent drop in the number of young men dying by suicide has taken place in Newcastle upon Tyne over the three decades analysed. Single young men show an increasing contribution to the total numbers of suicides. This increase is accompanied by a rise in the proportion living alone – social isolation has long been recognised as a significant factor associated with suicide.^9,10^ In spite of the increase of divorce among young men in the population, it is notable that they do not account for more deaths by suicide.

Previous studies have shown an association of suicide in young men with unemployment.^11^ In 1999 Gunnell *at al*^12^ found an association between suicide and general population unemployment rates, although they were not able to examine by age-specific unemployment rates. We have found an increasingly high proportion of unemployment in young men who died by suicide, present in two-thirds by the 2000s (period C), compared with previous studies which found rates of 39%^13^ and 36%.^14^ This is perhaps not surprising when one considers the effect of being unemployed at a time when young men are normally establishing their economic and social standing in society.

The significant reduction in use of carbon monoxide and barbiturates as a method of suicide from period A to period B coincided with the abolition in 1972 of domestic coal gas in Newcastle and the virtual withdrawal of barbiturates from clinical practice, emphasising the importance of minimising the availability of methods to help reduce suicide.^1,15^ Suicides using carbon monoxide from car exhausts remained prevalent in the 1990s but the compulsory introduction of catalytic converters (which reduce carbon monoxide content from car exhausts) has strikingly reduced these fatalities by period C, as it also appears to have happened elsewhere.^16–18^ Equally striking is the significant rise in suicides by hanging,^19^ although Biddle *et al*^18^ observed a decrease in hanging emerging in young men in the 21st century after a period of steady rise previously.

It is interesting to note that the proportion and type of cases of antidepressant misuse has increased despite the introduction of relatively safer drugs. Not all antidepressants will have been prescribed for depression but the assessment of suicide intent in patients with depression and a need to prescribe safely are important.^20^ Guidelines for depression in the UK exist to help practitioners enquire about suicide, stating that ‘not asking about suicide in someone depressed is like not asking about allergies when prescribing’ (information available from the author on request).

Paracetamol had previously been frequently used in suicide but in September 1998 the Royal Pharmaceutical Society of Great Britain limited the quantities which may be obtained without prescription. This restriction has reduced suicide and liver complications due to paracetamol overdose in the UK^15^ overall, and in Newcastle by the 2000s (period C) we noted only six cases of suicide involving paracetamol, of which in four cases it was taken with another substance. Indeed, an interesting feature in this study was the number of individuals who took a variety of drugs available to them and thus provided a combination that ensured lethality. This determination to die is poignantly expressed in individuals who combine an unequivocal act such as hanging with a massive drug overdose.

The relatively high incidence of jumping from heights and drowning is likely to be related to the opportunities afforded by a number of bridges that span the River Tyne. Strategies need to be employed to erect preventive measures at these ‘hot-spots’, and indeed since 2007 there have been safety notices displayed on Newcastle's high bridges, which has resulted in reduced calls to police negotiators.^21^

We have found an almost doubling of suicide associated with alcohol from the 1960s to the last two decades we studied, present in period C in over 40% of cases. In Lothian, Scotland, of men aged 20–29 who died by suicide, just over a third had alcohol detected at autopsy and 26% were above the legal limit for driving.^22^ In England and Wales, a third of men under age 25 who died by suicide were intoxicated at the time of death.^23^ Our study supports the importance not only of tackling alcohol misuse as a risk factor for premature death,^24,25^ but also of educating the population regarding the danger of alcohol in someone with an already depressed mood.

Most precipitating factors were similar in frequency in the periods studied, with the exemption of ‘failure of a long-term relationship’, which increased over time, and it is surprising that criminal justice involvement and housing issues have in fact decreased in frequency.

Around half the men in the first two periods and over three quarters in period C had at some time received treatment for mental health problems. Appleby *et al*^14^ found 76% of people aged under 35 who had died by suicide had a psychiatric history, including 45% who were under mental health services at the time of suicide compared with 3% of controls.^14^ Understanding why these individuals choose to die rather than utilise services at the time of ultimate despair should be a priority for commissioners and practitioners alike. It offers some reassurance that we found no individuals waiting for psychiatric admission during any period, considering the closure of many psychiatric in-patient facilities over recent decades. It is interesting to see that the proportion of those treated as an in-patient fell over the three periods studied and the proportion receiving out-patient treatment increased. This would seem to mirror the changing practice in psychiatry services from hospital- to community-focused care. Our study did not address the timing of their last primary care consultation. Vassilas & Morgan^26^ found consultation rates with GPs in the 4 weeks before suicide to be significantly lower in people aged under 35 compared with those aged over 35 (20% and 48% respectively).

Our findings show a significant increase over time in individuals with a history of a suicide attempt; present in 67% of cases in period C, which compares with 45%,^13^ 68%^14^ and 50%^22^ noted elsewhere, highlighting the continued need for specialist psychosocial assessments after self-harm.^27^

A report found that in nearly 10% of deaths by suicide (of people of all ages) a chronic or terminal physical health condition may have been a contributory factor.^28^ We found similar proportions of young males had an established physical illness during the first two study periods but only one person in period C did and the proportion of suicide in which physical illness was identified as a precipitating factor fell. It is difficult to explain this and echo calls for research in this area. We have previously reported that a high percentage of people who died by suicide in County Durham had seen their GP for low back pain in the 3 months preceding their death.^29^

### Study strengths and limitations

Our data are derived from coroner's records, which is an established and reliable method of identifying suicide cases, provided cases with an ‘open verdict’ are also examined and if appropriate, included in the suicide group.^5^ The nature of our investigation did not allow us to check psychiatric or GP records, although coroner's records frequently included medical reports. Data relating to the mental or physical health of deceased persons were thus based on coroner's records only and may therefore underestimate certain variables as described earlier. A further extension to this research may be to cross-check our data with other available sources of medical information. In a few cases, post-mortem alcohol levels were not available which may slightly underestimate the number of cases where alcohol was consumed prior to suicide.

Regrettably, we were not able to obtain age-specific data for Newcastle upon Tyne regarding unemployment, marital status or those living alone for all three study periods. As such, we have not been able to calculate age-specific suicide rates for these categories.

### Key challenges

Suicide prevention remains the raison d'être of suicide research. Our findings point to an approach specifically targeted at young males who, as Keith Hawton so aptly put it, die ‘by their own young hand’.^30^ The National Suicide Prevention Strategy for England and Wales^31^ is tackling high-risk groups but local action is required to drive forward initiatives to reduce suicide. These need to be multifaceted and dynamic, as our study clearly demonstrates that characteristics of young males who die by suicide change over time. Specifically we would recommend, based on our study findings:

- improvement of safety of medication and safety measures at sites of possible suicide needs to continue, but the lethality of hanging is a factor that cannot be as readily influenced
- strategies to reduce the association of suicide with alcohol in young men are urgently required
- better understanding of the support needed by young men going through a relationship crisis
- mental health services should evaluate why individuals dying by suicide are choosing not to utilise mental health services
- other services, such as general practice,^32,33^ police,^34^ ambulance and Social Services see these young men in the months before suicide and require training to help identify and modify risk; equally, services managing chronic or terminal physical illnesses may need to consider when and how to evaluate suicide risk.^28^

Although a number of changes seen in young males who die by suicide have been highlighted, examination of individual case histories equally demonstrates that suicide frequently results from a combination of factors. Understanding the complexity and nature of interacting factors leading to vulnerability, risk and premature death from suicide for young men remains a challenge for researchers.
